# Antibiotic Exposure Has Sex-Dependent Effects on the Gut Microbiota and Metabolism of Short-Chain Fatty Acids and Amino Acids in Mice

**DOI:** 10.1128/mSystems.00048-19

**Published:** 2019-06-04

**Authors:** Hongchang Gao, Qi Shu, Jiuxia Chen, Kai Fan, Pengtao Xu, Qi Zhou, Chen Li, Hong Zheng

**Affiliations:** a Institute of Metabonomics & Medical NMR, School of Pharmaceutical Sciences, Wenzhou Medical University, Wenzhou, China; University of California, San Diego

**Keywords:** amino acid metabolism, antibiotic, microbiome, sex-dependent effect, short-chain fatty acid

## Abstract

The gut microbiota has the capability to regulate homeostasis of the host metabolism. Since antibiotic exposure can adversely affect the microbiome, we hypothesized that antibiotic effects on the gut microbiota and host metabolism are sex dependent. In this study, we examined the effects of antibiotic treatments, including vancomycin (Vanc) and ciprofloxacin-metronidazole (CiMe), on the gut microbiome and metabolome in colonic contents and tissues in both male and female mice. We found that the relative abundances and structural composition of *Firmicutes* were significantly reduced in female mice after both Vanc and CiMe treatments but in male mice only after treatment with Vanc. However, Vanc exposure considerably altered the relative abundances and structural composition of representatives of the *Proteobacteria* especially in male mice. The levels of short-chain fatty acids (SCFAs; acetate, butyrate, and propionate) in colonic contents and tissues were significantly decreased in female mice after both antibiotic treatments, while these reductions were detected in male mice only after Vanc treatment. However, another SCFA, formate, exhibited the opposite tendency in colonic tissues. Both antibiotic exposures significantly decreased the levels of alanine, branched-chain amino acids (BCAAs; leucine, isoleucine, and valine) and aromatic amino acids (AAAs; phenylalanine and tyrosine) in colonic contents of female mice but not in male mice. Additionally, female mice had much greater correlations between microbe and metabolite than male mice. These findings suggest that sex-dependent effects should be considered for antibiotic-induced modifications of the gut microbiota and host metabolism.

**IMPORTANCE** Accumulating evidence shows that the gut microbiota regulates host metabolism by producing a series of metabolites, such as amino acids, bile acids, fatty acids, and others. These metabolites have a positive or negative effect on host health. Antibiotic exposure can disrupt the gut microbiota and thereby affect host metabolism and physiology. However, there are a limited number of studies addressing whether antibiotic effects on the gut microbiota and host metabolism are sex dependent. In this study, we uncovered a sex-dependent difference in antibiotic effects on the gut microbiota and metabolome in colonic contents and tissues in mice. These findings reveal that sex-dependent effects need to be considered for antibiotic use in scientific research or clinical practice. Moreover, this study will also give an important direction for future use of antibiotics to modify the gut microbiome and host metabolism in a sex-specific manner.

## Abstract (precis)

Accumulating evidence shows that the gut microbiota regulates host metabolism by producing a series of metabolites, such as amino acids, bile acids, fatty acids, and others. These metabolites have a positive or negative effect on host health. Antibiotic exposure can disrupt the gut microbiota and thereby affect host metabolism and physiology. However, there are a limited number of studies addressing whether antibiotic effects on the gut microbiota and host metabolism are sex dependent. In this study, we uncovered a sex-dependent difference in antibiotic effects on the gut microbiota and metabolome in colonic contents and tissues in mice. These findings reveal that sex-dependent effects need to be considered for antibiotic use in scientific research or clinical practice. Moreover, this study will also give an important direction for future use of antibiotics to modify the gut microbiome and host metabolism in a sex-specific manner.

## INTRODUCTION

The gut microbiota plays a key role in regulation of the host metabolism (1, 2). A series of metabolites can be produced by the gut microbiota, such as amino acids, bile acids, fatty acids, lipids, vitamins, and choline metabolites (1). Of note, these metabolites may have a positive or negative effect on host health. For example, short-chain fatty acids (SCFAs), fermented from dietary fiber, possess beneficial effects on the host, involving inflammation (3), type 1 diabetes (T1D) (4), and lipid metabolism (5). However, trimethylamine-N-oxide (TMAO), as a microbial metabolite of dietary choline, has been reported to promote arteriosclerosis (6), chronic kidney disease (7), and thrombosis (8). Thus, exploring host-microbe metabolic interactions will advance the understanding of host health and disease (2).

The composition of the gut microbiota is highly variable and significantly affected by many factors, such as age, genetics, environment, and diet (9). In addition, antibiotic treatment, as an important therapeutic intervention, can also result in the disturbance of the gut microbiota and further influence host metabolism and physiology (10, 11). Theriot et al. (12) reported that antibiotic (cefoperazone) use induced considerable changes in the gut microbiome and in turn converted the metabolic environment that facilitates the germination and growth of Clostridium difficile. Antibiotic treatment (ciprofloxacin) can cause alterations in the host metabolic environment, potentially giving rise to impaired drug efficacy and immune function (13). Livanos et al. (14) revealed that early-life antibiotic treatment (penicillin V potassium salt and macrolide tylosin tartrate) altered the gut microbiome, host metabolism, gene expression, and T-cell populations, accelerating the development of T1D in mice. In addition, gender effects on the gut microbiota and host physiology have also received much attention. For example, Markle et al. (15) found that a fecal transplant from male to female obviously changed the recipient’s microbiota and metabolome, increased the testosterone level, alleviated islet inflammation, and protected against T1D development in nonobese diabetic mice. Moreover, sex differences in the gut microbiota also impacted host autoimmunity (16) and brain microglial properties (17). However, little information is available regarding the interaction effect between sex and antibiotic on the gut microbiota and host metabolome.

In the present study, we treated both male and female mice with broad-spectrum and nonabsorbable antibiotics: vancomycin (Vanc), targeting Gram-positive bacteria, and ciprofloxacin-metronidazole (CiMe), targeting Gram-positive bacteria (18). Gut microbiota composition in colonic contents was analyzed by 16S rRNA gene sequencing, and metabolomic profiles in colonic contents and tissues were measured using nuclear magnetic resonance (NMR) spectroscopy. A generalized linear mixed model (GLMM) was subsequently used to evaluate the interaction effect of sex and antibiotic on the gut microbiota and metabolite. The relationship between microbe and metabolite was further analyzed by Pearson correlation and network analysis. The aims of this study were (i) to identify the microbe and metabolite that had a significant gender-antibiotic interaction effect and (ii) to investigate sex differences in the host-microbe metabolic interaction.

## RESULTS

### Sex-dependent microbial composition changes in mice.

**FIG 1:**
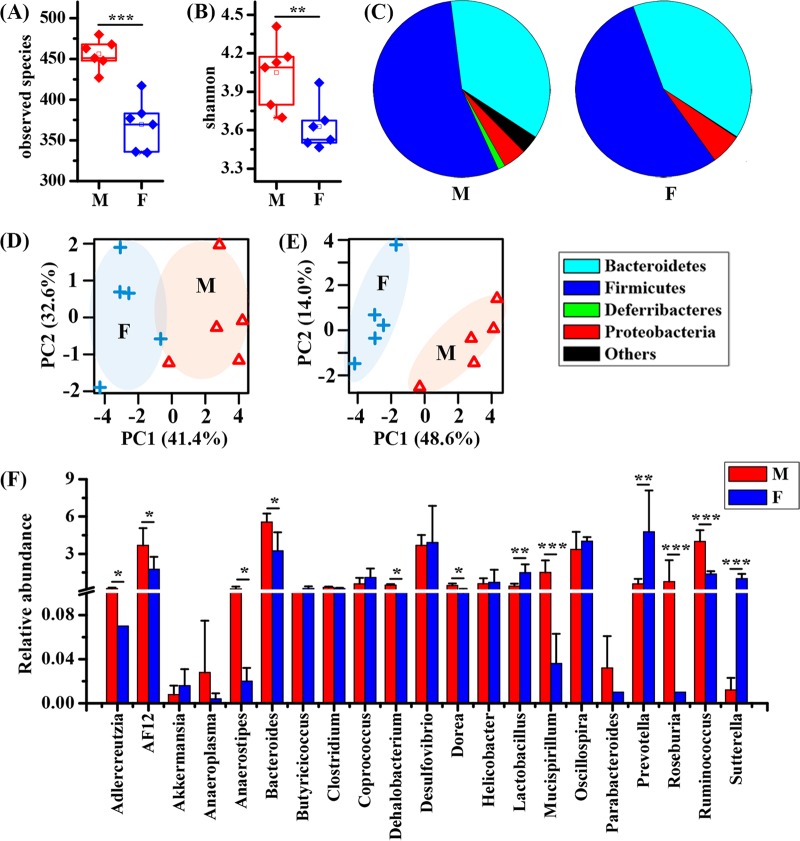
Sex differences in the gut microbiota of healthy mice. (A) Observed species. (B) Shannon index. (C) The percentages of the microbial composition at the phylum level. (D) Classification based on PCA using the gut microbiome at the phylum level. (E) Classification based on PCA using the gut microbiome at the genus level. (F) Relative abundance of the microbial composition at the genus level. Pairwise comparisons were analyzed using Student's *t* test with a Bonferroni adjustment. M, male; F, female. Significance level: *, *P* < 0.05; **, *P* < 0.01; ***, *P* < 0.001.

The microbiome in colonic contents was analyzed by 16S rRNA gene sequencing, and the alpha diversity was calculated in both male and female mice (Fig. 1). The observed species and taxon diversity (Shannon index) of the gut microbiota were significantly decreased in female mice relative to levels in male mice, as shown in Fig. 1A and B, respectively. At the phylum level, female mice possessed obviously decreased proportions of *Firmicutes* and *Deferribacteres* as well as increased proportions of *Bacteroidetes* and *Proteobacteria* compared to levels in male mice (Fig. 1C). Principal-coordinate analysis (PCA) results based on the gut microbiome also show clear separations between male and female mice at both the phylum (Fig. 1D) and genus (Fig. 1E) levels. Furthermore, relative to levels in male mice, we found that the abundances of *Adlercreutzia*, *AF12*, *Anaeroplasma*, *Bacteroides*, *Dehalobacterium*, *Dorea*, *Mucispirillum*, *Roseburia*, and *Ruminococcus* were significantly reduced in female mice (Fig. 1F). However, female mice had higher abundances of *Lactobacillus*, *Prevotella*, and *Sutterella* than male mice.

### Sex-dependent metabolic changes in mice.

**FIG 2:**
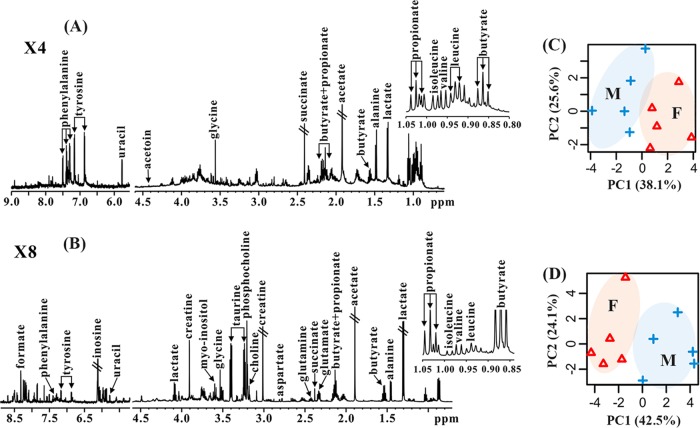
Sex differences in the metabolomic profile of healthy mice. Typical 600 MHz ^1^H NMR spectra of colonic contents (A) and colonic tissues (B) of mice are shown. A classification based on PCA was performed using the metabolomic profile in colonic contents (C) and colonic tissues (D) of mice. M, male; F, female. X4, 4 times magnification; X8, 8 times magnification.

Metabolomic profiles in colonic contents and tissues of mice were analyzed using NMR spectroscopy, and the corresponding NMR spectra are illustrated in Fig. 2A and B, respectively. In total, 24 metabolites were identified from NMR-based metabolomic analysis, mainly including short-chain fatty acids (SCFAs) (formate, acetate, butyrate, and propionate), amino acids (alanine, leucine, isoleucine, valine, glutamate, glutamine, aspartate, glycine, tyrosine, phenylalanine, and taurine), energy metabolism (succinate, creatine, and lactate), choline metabolism (choline and phosphocholine), and others (acetoin, myo-inositol, uracil, and inosine).

**FIG 3:**
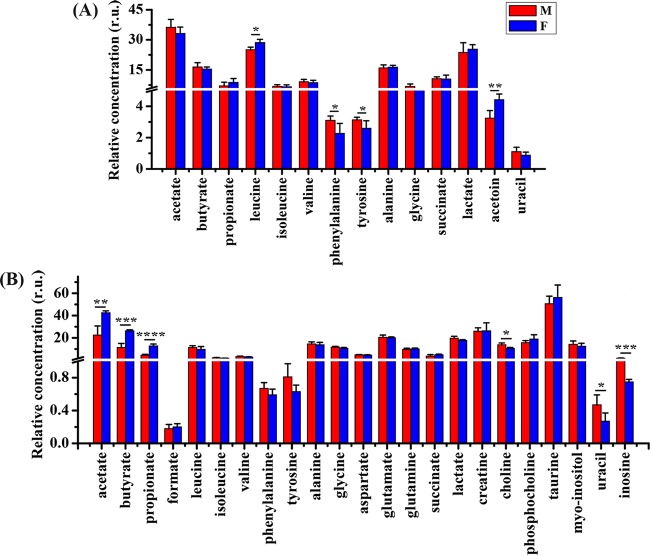
Sex-dependent metabolic differences in healthy mice. (A) Metabolic changes in colonic contents. (B) Metabolic changes in colonic tissue. Pairwise comparisons were analyzed using Student's *t* test with a Bonferroni adjustment. M, male; F, female; r.u., relative units. Significance level: *, *P* < 0.05; **, *P* < 0.01; ***, *P* < 0.001; ****, *P* < 0.0001.

PCA results show that clear discriminations between male and female mice were observed in the metabolome of both colonic contents (Fig. 2C) and tissues (Fig. 2D). In addition, as can been seen from Fig. 3A, the levels of leucine and acetoin in colonic contents were significantly increased in female mice relative to levels in male mice, while the opposite changes were detected in phenylalanine and tyrosine. In colonic tissues, however, male mice had lower levels of acetate, butyrate, and propionate as well as higher levels of choline, uracil, and inosine than female mice (Fig. 3B).

### Sex differences in antibiotic effects on the microbial diversity and composition in mice.

**FIG 4:**
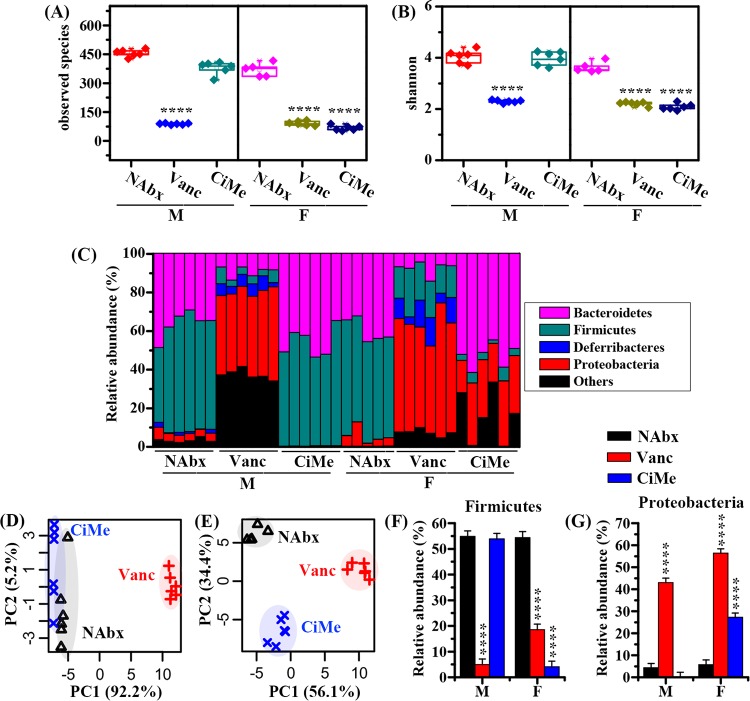
Sex differences in antibiotic effects on the gut microbiota at the phylum level in healthy mice. (A and B) Changes in the observed species and Shannon index in mice after antibiotic exposures. (C) Relative abundance of the microbial composition after antibiotic exposure. A classification based on PCA was performed using the gut microbiome in male (D) and female (E) mice. (F and G) Changes in abundances of *Firmicutes* and *Proteobacteria* in mice after antibiotic exposure. The interaction effect between sex and antibiotic on microbes was evaluated by a linear mixed-model, and pairwise multiple comparisons were analyzed using Student's *t* test with a Bonferroni adjustment. NAbx, no antibiotic exposure; Vanc, vancomycin; CiMe, ciprofloxacin and metronidazole; M, male; F, female. Significance level: ****, *P* < 0.0001.

To study sex-dependent effects of antibiotic exposure on changes in microbial diversity, the microbiome in colonic contents of both male and female mice was analyzed after 14 days of antibiotic treatments, and alpha diversity was assessed for non-antibiotic-treated (NAbx) and antibiotic-treated (Vanc and CiMe) groups. The taxon richness (observed species) and diversity (Shannon index) of the gut microbiota are shown in Fig. 4A and B, respectively. Both antibiotic treatments resulted in significant reductions in taxon richness (Fig. 4A) and diversity (Fig. 4B) in female mice compared with levels in the NAbx group. However, for male mice, taxon richness and diversity were significantly decreased only after Vanc treatment, as illustrated in Fig. 4A and B, respectively.

Figure 4C illustrates a detailed overview of the microbial composition profile in each mouse at the phylum level. In female mice, we found that after both Vanc and CiMe treatments, the microbiota composition was obviously different from that of the NAbx group (Fig. 4C). Yet a distinct difference in microbiota composition was detected only in male mice treated with Vanc. Subsequently, PCA was conducted to further examine the discrimination between different groups based on the microbiota composition. For male mice, Vanc treatment was clearly separated from the other two groups along the PC1 axis, while NAbx and CiMe groups were close to each other, as shown in Fig. 1D. However, the distinction in the microbial composition patterns arising from both Vanc and CiMe exposures can be clearly seen from the PCA score plot in Fig. 4E for female mice.

In this study, the interaction effect of sex and antibiotic on the gut microbiota was assessed by the GLMM analysis. Only *Firmicutes* and *Proteobacteria* showed significant interaction effects, as illustrated in Fig. 4F and G, respectively. Both antibiotic treatments resulted in significant reductions in the relative abundances of *Firmicutes* in female mice (*P* < 0.0001), but in male mice a significant decrease was found only after Vanc treatment (*P* < 0.0001). For the *Proteobacteria* phylum, Fig. 4G shows that high abundances were detected in both male and female mice after Vanc exposure relative to those in the NAbx group (*P* < 0.0001). Yet after CiMe treatment, *Proteobacteria* relative abundance was significantly increased in female mice but not in male mice (Fig. 4G).

**FIG 5:**
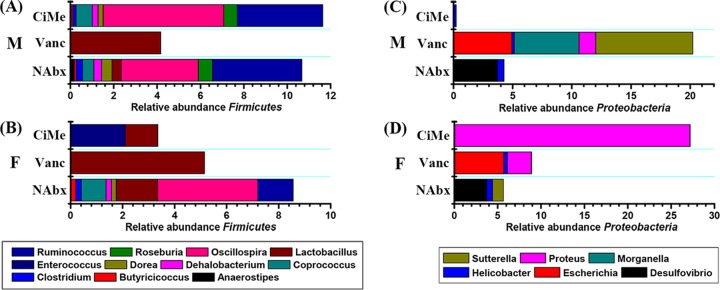
Sex differences in antibiotic effects on the microbial composition at the genus level in the *Firmicutes* and *Proteobacteria* phylum in healthy mice. (A) Relative abundance of the major microbial genus in the *Firmicutes* phylum in male mice. (B) Relative abundance of the major microbial genus in the *Firmicutes* phylum in female mice. (C) Relative abundance of the major microbial genus in the *Proteobacteria* phylum in male mice. (D) Relative abundance of the major microbial genus in the *Proteobacteria* phylum in female mice. NAbx, no antibiotic exposure; Vanc, vancomycin; CiMe, ciprofloxacin and metronidazole; M, male; F, female.

**FIG 6:**
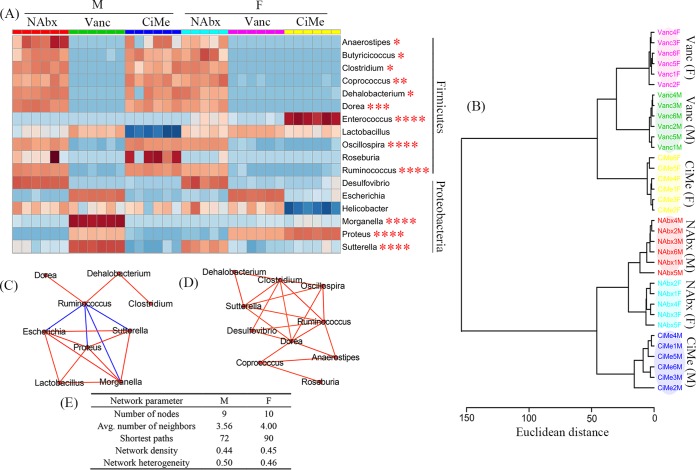
Sex differences in antibiotic effects on the gut microbiota at the genus level in healthy mice. (A) Heat map showing relative abundances of the microbial composition in the *Firmicutes* and *Proteobacteria* phyla after antibiotic exposure. (B) Cluster analysis based on the gut microbiota by Ward’s method and Euclidean distance. (C and D) Correlation networks between different microbes in male (C) and female (D) mice (|*r*| > 0.80; red line, positive correlation; blue line, negative correlation) and the main network topological parameters (E). NAbx, no antibiotic exposure; Vanc, vancomycin; CiMe, ciprofloxacin and metronidazole; M, male; F, female. Significance level: *, *P* < 0.05; **, *P* < 0.01; ***, *P* < 0.001; ****, *P* < 0.0001.

Figure 5 illustrates an overview of the microbial composition at the genus level from the *Firmicutes* and *Proteobacteria* phyla, and the corresponding statistical results from the GLMM analysis are shown in Fig. 6A. For the *Firmicutes* phylum, genus-level structural composition was obviously altered in male mice treated only with Vanc (Fig. 5A) and in female mice after both Vanc and CiMe treatments (Fig. 5B). Moreover, Vanc exposure considerably varied and increased the genus-level structural composition of *Proteobacteria* especially in male mice (Fig. 5C). A dramatic increase in *Proteus* was detected in female mice treated with CiMe (Fig. 5D).

As can be seen from Fig. 6A, significant interaction effects between sex and antibiotic were identified for *Anaerostipes*, *Butyricicoccus*, *Clostridium*, *Coprococcus*, *Dehalobacterium*, *Dorea*, *Enterococcus*, *Oscillospira*, and *Ruminococcus* in the *Firmicutes* phylum as well as *Morganella*, *Proteus*, and *Sutterella* in the *Proteobacteria* phylum. The relative abundances of *Anaerostipes*, *Butyricicoccus*, *Clostridium*, *Coprococcus*, *Dehalobacterium*, *Dorea*, *Oscillospira*, and *Ruminococcus* were significantly decreased in female mice after both Vanc and CiMe exposures, but for male mice only Vanc treatment resulted in reductions. In female mice, high relative abundances were detected for *Enterococcus* and *Proteus* after CiMe treatment, but in male mice, we found high relative abundances of *Morganella*, *Sutterella*, and *Proteus* after Vanc treatment (Fig. 6A).

In this study, cluster analysis was further used to evaluate similarity between different groups based on the gut microbiota at the genus level without any a priori hypotheses (Fig. 6B). We found that male and female mice without antibiotic treatment (NAbx) were, as expected, clustered close to each other. In addition, Fig. 6B shows that genus-level microbial compositions were similar between male and female mice treated with Vanc. Interestingly, after CiMe treatment, female mice were more similar to mice treated with Vanc, but male mice did not seem to be affected by CiMe (Fig. 6B). Genus-level network analyses are presented in Fig. 6C for male mice and in Fig. 6D for female mice, and the main network topological parameters are listed in Fig. 6E. Relative to male mice, female mice had a high average number of neighbors (4.00 versus 3.56) and more shortest paths (90 versus 72). However, the number of nodes (10 versus 9), network density (0.45 versus 0.44), and heterogeneity (0.46 versus 0.50) were very close to each other (Fig. 6E). These results suggest that interrelations between microbes may have been stronger in female mice than in male mice. The detailed relationships were also shifted; for example, *Dorea* was correlated only with *Ruminococcus* in male mice (Fig. 6C), but in female mice *Dorea* had close associations with *Anaerostipes*, *Clostridium*, *Coprococcus*, *Oscillospira*, *Ruminococcus*, *Sutterella*, and *Desulfovibrio* (Fig. 6D).

### Sex differences in antibiotic effects on the metabolome in colonic contents and tissues of mice.

**FIG 7:**
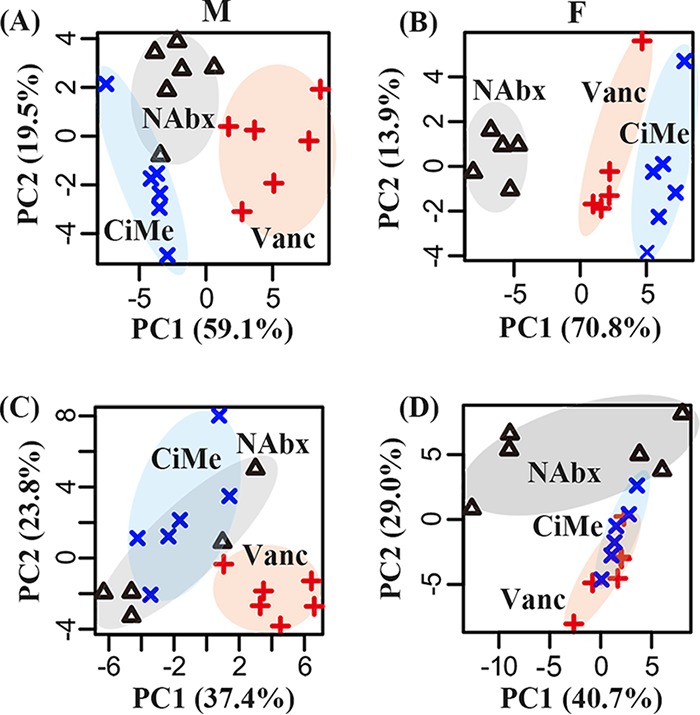
Classification based on PCA using the metabolomic profile in mice after antibiotic exposure. (A) Colonic contents in male mice. (B) Colonic contents in female mice. (C) Colonic tissues in male mice. (D) Colonic tissues in female mice. NAbx, no antibiotic exposure; Vanc, vancomycin; CiMe, ciprofloxacin and metronidazole; M, male; F, female.

The overview of metabolic pattern changes in both male and female mice after antibiotic exposures was analyzed by PCA and illustrated in a score plot, as shown in Fig. 7. For male mice, the Vanc group can be clearly separated from the other two groups based on the metabolome of colonic contents (Fig. 7A) and tissues (Fig. 7C), but the CiMe group was close to the NAbx group. Conversely, for female mice, the CiMe group was clearly distinguished from the NAbx group and close to the Vanc group, as shown in Fig. 7B for colonic contents and in Fig. 7D for colonic tissues. These findings were consistent with cluster analysis derived from the gut microbiome (Fig. 6B).

**FIG 8:**
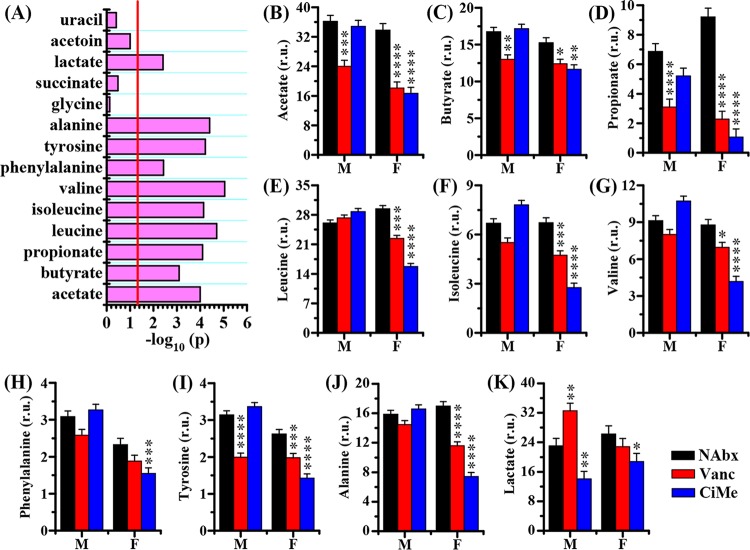
Sex differences in antibiotic effects on the metabolic level in colonic contents of healthy mice. (A) Statistical results of the interaction effect between sex and antibiotic on the metabolic changes. The interaction effect was evaluated by a linear mixed-model, and –log_10_(*P*) of >1.30 was considered statistically significant. (B to K) Changes in levels of acetate, butyrate, propionate, leucine, isoleucine, valine, phenylalanine, tyrosine, alanine, and lactate, as indicated, in colonic contents of mice after antibiotic exposure. Pairwise comparisons were analyzed using Student's *t* test with a Bonferroni adjustment. NAbx, no antibiotic exposure; Vanc, vancomycin; CiMe, ciprofloxacin and metronidazole; M, male; F, female. r.u., relative units. Significance level: *, *P* < 0.05; **, *P* < 0.01; ***, *P* < 0.001; ****, *P* < 0.0001.

**FIG 9:**
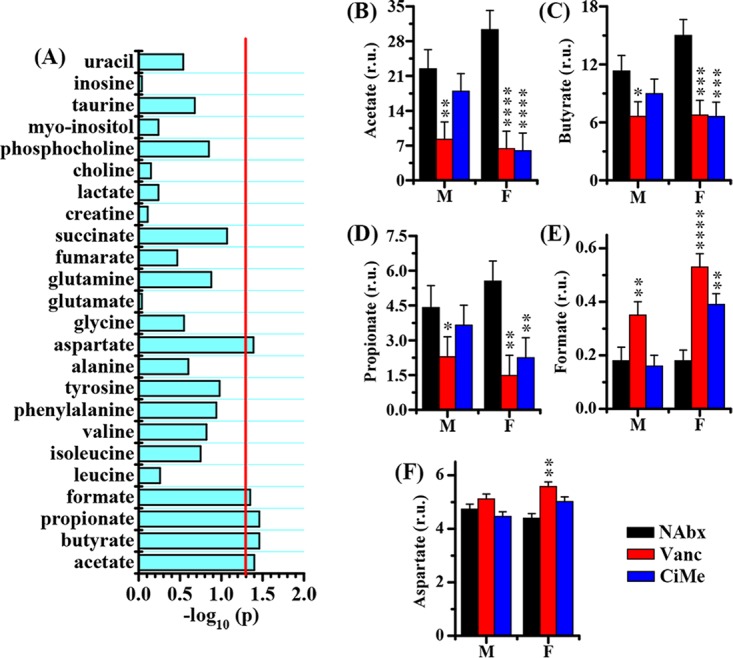
Sex differences in antibiotic effects on the metabolic level in colonic tissues of healthy mice. (A) Statistical results of the interaction effect between sex and antibiotic on metabolic changes. The interaction effect was evaluated by a linear mixed-model, and a –log_10_(*P*) of >1.30 was considered statistically significant. (B to F) Changes in levels of acetate, butyrate, propionate, formate, and aspartate in colonic tissues of mice after antibiotic exposure, as indicated. Pairwise comparisons were analyzed using Student's *t* test with a Bonferroni adjustment. NAbx, no antibiotic exposure; Vanc, vancomycin; CiMe, ciprofloxacin and metronidazole; M, male; F, female; r.u., relative units. Significance level: *, *P* < 0.05; **, *P* < 0.01; ***, *P* < 0.001; ****, *P* < 0.0001.

Then, a GLMM analysis was employed to evaluate the interaction effects between sex and antibiotic on the metabolic changes in mice. In colonic contents, we identified a series of metabolites that had significant interaction effects between sex and antibiotic, including acetate, butyrate, propionate, leucine, isoleucine, valine, phenylalanine, tyrosine, alanine, and lactate (Fig. 8A). However, significant interaction effects were detected only for acetate, butyrate, propionate, formate, and aspartate in colonic tissues, as shown in Fig. 9A. The levels of SCFAs in colonic contents and tissues, including acetate (Fig. 8B and 9B), butyrate (Fig. 8C and 9C), and propionate (Fig. 8D and 9D), were significantly decreased in female mice after both Vanc and CiMe exposures, but these reductions were found in male mice treated only with Vanc. In female mice, both Vanc and CiMe treatments gave rise to significantly decreased levels of branched-chain amino acids ([BCAAs] leucine, isoleucine, and valine) (Fig. 8E to G, respectively) and aromatic amino acids ([AAAs] phenylalanine and tyrosine) (Fig. 8H and I, respectively) in colonic contents. Yet, compared with levels in the NAbx group, a significant reduction in the tyrosine level of colonic contents was detected in male mice only after Vanc treatment (Fig. 8I) (P < 0.0001). Additionally, there were no significant interaction effects for BCAAs and AAAs in colonic tissues. Figure 8J shows that female mice, but not male mice, had a significant reduction in alanine levels in colonic contents after both antibiotic treatments. Moreover, relative to levels in the NAbx group, the level of lactate in colonic contents was significantly increased in male mice after Vanc exposure but significantly decreased after CiMe treatment in both types of mice (Fig. 8K). In colonic tissues, we found that Vanc treatment significantly increased the level of formate in both male and female mice; however, in female mice, a significant increase in formate level was also obtained after CiMe exposure (Fig. 9E). The level of aspartate in colonic tissues was significantly increased in female mice treated with Vanc, while no significant changes were observed in other groups (Fig. 9F).

### Sex differences in the host-microbe metabolic interaction in mice.

**FIG 10:**
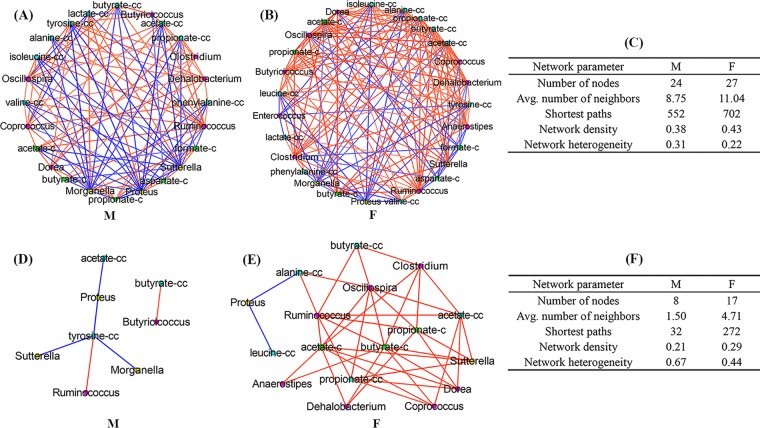
Correlation between the gut microbiota and metabolites. Correlation networks in male (A) and female (B) mice (|*r*| > 0.30) are shown, along with the corresponding network topological parameters (C). Correlation networks in male (D) and female (E) mice (|*r*| > 0.80) are shown along with the corresponding network topological parameters (F). The suffixes -c and -cc indicate metabolites detected in colonic tissues and colonic contents, respectively. Red line, positive correlation; blue line, negative correlation. M, male; F, female.

To explore the association between microbe and metabolite, a correlation matrix was assessed using the Pearson’s correlation analysis and presented as the correlation network shown in Fig. 10A for male mice and in Fig. 10B for female mice (|*r*| > 0.30), based on 12 bacterial genera and 12 metabolites that exhibited significant interaction effects between sex and antibiotic. The main topological parameters for the correlation network in male and female mice (|*r*| > 0.30) are presented in Fig. 10C; we found higher values for the number of nodes (27 versus 24), average number of neighbors (11.04 versus 8.75), number of shortest paths (702 versus 552), and network density (0.43 versus 0.38) in female mice than in male mice.

The strong relationships between microbe and metabolite (|*r*| > 0.80, *P* < 0.05) are illustrated in Fig. 10D for male mice and in Fig. 10E for female mice. Figure 10F shows that female mice had a much higher number of nodes (17 versus 8), average number of neighbors (4.71 versus 1.50), number of shortest paths (272 versus 32), and network density (0.29 versus 0.21) than male mice, suggesting that the host-microbe metabolic interaction in male mice became weaker than that in female mice. In female mice, acetate, butyrate, and propionate in colonic contents and tissues were mainly positively correlated with gut microbes in members of the *Firmicutes* phylum, including *Anaerostipes*, *Coprococcus*, *Clostridium*, *Oscillospira*, *Dorea*, *Dehalobacterium*, and *Ruminococcus* (Fig. 10E). However, in male mice, acetate and butyrate in colonic contents were identified to be correlated only with *Proteus* and *Butyricicoccus*, respectively (Fig. 10D). A negative relationship between *Proteus* and leucine was found in colonic contents of female mice; moreover, alanine showed a negative correlation with *Proteus* and was positively associated with *Ruminococcus* and *Oscillospira*. Figure 10D shows that tyrosine in colonic contents of male mice was negatively correlated with *Morganella* and *Sutterella* and positively correlated with *Ruminococcus*.

## DISCUSSION

The gut microbiota has the capability to generate a series of metabolites and thereby regulate homeostasis of the host metabolism (1). Antibiotic treatment, as an important therapeutic approach in clinical practice, can adversely affect the gut microbiota, inevitably giving rise to metabolic disorders (19). However, whether antibiotics have sex-dependent effects on the gut microbiota and host metabolism is unknown.

In the present study, at first we found that the gut microbiome and metabolome are influenced by gender. Results showed that male mice had higher abundances of *Adlercreutzia*, *AF12*, *Anaeroplasma*, *Bacteroides*, *Dehalobacterium*, *Dorea*, *Mucispirillum*, *Roseburia*, and *Ruminococcus* as well as lower levels of *Lactobacillus*, *Prevotella*, and *Sutterella* than female mice. In fact, sex-dependent differences in the gut microbiota have been reported due to hormonal effects (20), but many other factors may also affect microbial composition changes, such as genotype (21), age (22), body weight (23), and even diet (24). Thus, the impact of gender on the gut microbiome is a complex issue; however, of note, the gut flora may mediate sex differences in host physiology and disease (25), including autoimmunity (16), neurogenesis (17), liver cancer (26), and cardiovascular disease (27). Sex-dependent differences in the gut microbiota also affect the host metabolism. For example, Baars et al. (28) reported that sex differences in lipid metabolism are modulated by the gut microbiota in mice. In our study, female mice showed higher levels of leucine and acetoin in colonic contents and of SCFAs (acetate, butyrate, and propionate) in colonic tissues. Also, in female mice, we observed lower levels of phenylalanine and tyrosine in colonic contents as well as of choline, uracil, and inosine in colonic tissues than in male mice. Although the causal relationships or sex-specific differences still need to be confirmed in further studies, especially in clinical practice, sex differences in the gut microbiome and metabolome will aid in the development of sex-based medicine.

Furthermore, our study also reveals that antibiotic exposure impacts the gut microbiota in a sex-specific manner. Relative to levels in the NAbx group, both antibiotic treatments significantly reduced microbial composition alpha diversity in female mice, but a significant decrease was detected in male mice only after Vanc treatment. Since *Firmicutes* is a major phylum of Gram-positive bacteria, Vanc exposure significantly reduced its relative abundance in both male and female mice. However, unexpectedly, the abundance of the *Firmicutes* phylum was also decreased in female mice after CiMe exposure, which was mainly attributed to decreases in the relative abundances of *Oscillospira*, *Coprococcus*, *Butyricicoccus*, *Clostridium*, *Dehalobacterium*, and *Dorea* at the genus level. In addition, we also surprisingly found that CiMe significantly increased the *Proteobacteria* in female mice, a major phylum of Gram-negative bacteria, due to a dramatic growth of *Proteus*. These findings indicate that the antibiotic effect on the gut microbiota was different in female mice from that in male mice, suggesting that antibiotic use in scientific research or clinical practice may need to consider gender as one influence factor. In this study, we did not further explore its potential reasons, but sex steroid hormones could be one of the primary causes (29).

Host-microbiota metabolic interactions are dynamic, so antibiotic-induced changes in the gut microbiota can result in the alteration of host metabolism (1). SCFAs, as key bacterial metabolites (30), would be the first to bear the brunt. We found that both antibiotic treatments significantly decreased the levels of SCFAs, including acetate, butyrate, and propionate, in colonic contents and in tissues of female mice, but these reductions were observed only in male mice treated with Vanc, which is consistent with microbial composition alterations. Moreover, of note, the level of formate, another SCFA, showed an opposite response to antibiotic exposures in colonic tissues. Correlation network analysis revealed that the gut microbiota was more closely related to SCFAs in female mice than in male mice. We found that SCFAs in both colonic contents and tissues of female mice had strong relationships with the gut microbiota, including *Anaerostipes*, *Coprococcus*, *Clostridium*, *Ruminococcus*, *Oscillospira*, *Dorea*, *Sutterella*, and *Dehalobacterium*. However, only two bacteria, *Butyricicoccus* and *Proteus*, were identified to be highly correlated with acetate and butyrate in colonic contents of male mice. In addition, the gut microbiota also has a key role in the supply of both BCAAs and AAAs to maintain host amino acid homeostasis (31, 32). For this reason, antibiotic-induced gut microbial composition modification inevitably results in disrupted amino acid metabolism. Interestingly, we found that both antibiotics, especially Vanc, significantly reduced the levels of alanine, BCAAs, and AAAs in colonic contents of female mice; however, antibiotic effects were not statistically significant in male mice, excepting a significantly decreased tyrosine level after Vanc treatment. These findings may imply the sex-specific effect of antibiotic exposure on bacterial protein fermentation or degradation. In this study, several associations were observed between the gut microbiota and amino acids in mice. The gut microbiota modulating host amino acid metabolism was also found in mice by Mardinoglu et al. (33). Moreover, Sridharan et al. (32) reported that amino acid metabolism was largely affected by the gut microbiota, of which *Proteobacteria* could be the most important moderator. Interestingly, we identified that *Proteus* in the *Proteobacteria* phylum had highly negative relationships with alanine and leucine in colonic contents of female mice and tyrosine in male mice. In addition, the tyrosine level in colonic contents of male mice was also strongly correlated with *Morganella* and *Sutterella* in the *Proteobacteria* phylum as well as with *Ruminococcus* in the *Firmicutes* phylum. Lactate and aspartate also showed sex-dependent responses to antibiotic treatments; for example, Vanc treatment significantly increased the lactate level in colonic contents of male mice and the aspartate level in colonic tissues of female mice. In our study, however, no strong relationships were identified between these two metabolites and microbes. Taken together, host metabolism, particularly for SCFAs, BCAAs, and AAAs, was altered by antibiotic-induced gut microbial composition modification in a sex-specific manner.

Although the link between the gut microbiota and host health is complex, bacterial metabolites such as SCFAs, BCAAs, and AAAs have been regarded as important modulators of host physiology (32). For example, SCFAs generally exert health-promoting effects, including suppressing inflammatory responses (34), regulating energy homoeostasis (35), enhancing insulin sensitivity (36), promoting metabolic benefits (37), and protecting against obesity (38). SCFAs can also improve colonic functions, such as reinforcement of the colonic barrier (39, 40) and modulation of colonic secretion (41). Yet another SCFA, formate, has been reported to promote intestinal inflammation (42). BCAAs belong to essential amino acids and have been reported as pharmacological nutrients for liver disease (43) and hepatic encephalopathy (44). In addition, AAAs, including tyrosine and phenylalanine, are also very important amino acids in the body for protein and neurotransmitter syntheses (45). However, for diabetes, these amino acids have adverse effects on insulin sensitivity (46–48). Wang et al. (49) reported that increases in BCAAs and AAAs may result in a 5-fold increased risk of developing type 2 diabetes mellitus (T2DM) in the future. Additionally, increased BCAA levels have also been shown to promote cancer (50), heart failure (51), and kidney disease (52). Aspartate, as a nonessential amino acid, plays an important role in mitochondrial respiration during cell proliferation (53, 54); however, aspartate has been reported as a limiting metabolite for tumor growth (55). Therefore, metabolites may have both positive and negative effects on physiological functions, suggesting that the impact of microbiota-mediated metabolic changes for human health and disease are complex. Additionally, it should be noted that metabolites can also be used as potential biomarkers of diseases, such as BCAAs and AAAs for diabetes (56), aspartate for breast cancer (57), and lactate for short-bowel syndrome (58, 59). In the current study, we propose that antibiotic-induced alterations in the gut microbiota would affect host metabolism in a sex-specific manner, which may result in different physiological effects between two genders and also highlights that sex-based differences in antibiotic responses need to be noticed for disease diagnosis using metabolic markers.

Overall, our results reveal that antibiotic exposure exhibits sex-dependent effects on the gut microbiome and host metabolism in mice. Therefore, more attention should be paid to sex differences in the effects of antibiotics on host physiology and diseases. However, these findings still need to be validated in clinical trials in order to better guide future usage of antibiotics in a sex-specific manner. In addition, further work should include studies analyzing the causal relationship between microbe and metabolite although the host-microbe metabolic interaction is extremely complex.

## MATERIALS AND METHODS

### Animals.

Male and female C57BL/6 mice aged 6 weeks (*n* = 30 for each gender), weighing approximately 20 g, were purchased from the SLAC Laboratory Animal Co., Ltd. (Shanghai, China). All mice were housed in a specific-pathogen-free (SPF) animal facility under constant conditions (temperature, 22 ± 1°C; humidity, 55% ± 5%; light-dark cycle, 12 h/12 h; lights on at 8:00 a.m.) at the Laboratory Animal Center of Wenzhou Medical University (WMU; Wenzhou, China) and given free access to standard rat chow and tap water. This study was conducted in line with the *Guide for the Care and Use of Laboratory Animals* (60) and approved by the Institutional Animal Care and Use Committee of WMU.

### Antibiotic treatment.

After a 2-week acclimation period, male and female mice were randomly divided into three groups: non-antibiotic-treated (NAbx) and vancomycin (Vanc)- and ciprofloxacin-metronidazole (CiMe)-treated groups. The control mice received drinking water without any antibiotics until the end of the antibiotic treatments. For the two antibiotic groups, antibiotics were purchased from Sigma-Aldrich and given to mice in their drinking water (vancomycin, 0.5 g/liter; ciprofloxacin, 0.2 g/liter; metronidazole, 1.0 g/liter).

### Gut microbiota analysis.

The microbial DNA in colonic contents was extracted by a Stool DNA Isolation kit according to the manufacturer’s instructions (TIANGen, China), and purity was detected using 1% agarose gel electrophoresis. The V4 region of the bacterial 16S rRNA gene was amplified with the barcoded primer pair 515F/806R (515F, 5′-GTG CCA GCM GCC GCG GTA A-3′; 806R, 5′-GGA CTA CHV GGG TWT CTA AT-3′). PCR products were detected by 2% agarose gel electrophoresis, purified with a QIAquick Gel Extraction kit (Qiagen, Germany), and finally sequenced on an Illumina HiSeq2500 PE250 sequencer (Illumina, San Diego, CA, USA) at Novogene (Beijing, China).

The sequencing data were subjected to a series of bioinformatics analyses. First, raw tags were generated from FLASH software (version 1.2.7) through merging paired-end reads (61), and clean tags were obtained by QIIME (version 1.7.0) analysis (62). Then, effective tags were obtained by the UCHIME algorithm (63) and clustered into operational taxonomic units (OTUs) with UPARSE software (version 7.0.1001) based on a similarity threshold of 97% (64). Representative sequences from OTUs were annotated with the taxonomic information in accordance with the Mothur method and SILVA SSUrRNA database (65). The alpha and beta diversities of the gut microbiota were analyzed by QIIME software (version 1.7.0) and R software (version 2.15.3).

### Sample collection and metabolite extraction.

After 2 weeks of antibiotic treatments, mice were sacrificed by rapid decapitation, and colonic contents and tissues were collected, frozen in liquid nitrogen promptly, and kept at −80°C until use. An aliquot of colonic contents (0.1 g) was weighed into an Eppendorf tube and diluted 5 times in phosphate-buffered saline (PBS; pH 7.4). The mixture was extracted with ultrasonic-assisted extraction for 15 min and centrifuged at 5,000 × *g* at 4°C for 15 min. Finally, 400 μl of supernatant was transferred into an NMR tube and mixed with 100 μl of D_2_O containing 0.05% of sodium trimethylsilyl propionate-d_4_ (TSP; 0.42 mM) for NMR analysis. For colonic tissues, an aliquot of sample (0.1 g) was weighed into an Eppendorf tube and 4 ml/g of ice-cold methanol and 0.85 ml/g of ice-cold water were added. The tissue sample was ground by using an electric homogenizer (Fluko, Shanghai, China) and mixed with 2 ml/g of ice-cold chloroform and 2 ml/g of ice-cold water. After blending, the mixture was homogenized using a vortex mixer, placed on ice for 15 min, and centrifuged at 10,000 × *g* at 4°C for 15 min. Then, the supernatant was transferred into a new Eppendorf tube and lyophilized for 24 h. The dried powder was redissolved in 0.6 ml of D_2_O (0.05% TSP) and transferred into an NMR tube for NMR measurement.

### NMR-based metabolomic analysis.

^1^H NMR spectra were recorded using a Bruker Avance III 600 spectrometer (Bruker BioSpin, Rheinstetten, Germany). The standard single-pulse sequence, ZGPR, with water signal presaturation was performed to determine metabolomic profiles in colonic contents and tissues at 25°C. The main acquisition parameters were set as follows: acquisition time, 2.66 s/scan; data points, 256 K; spectral width, 12,000 Hz; relaxation delay, 4 s.

The NMR spectra were manually corrected for phase/baseline and referenced to TSP peak at 0 ppm by using Topspin, version 3.0, software (Bruker BioSpin, Rheinstetten, Germany). Then, the icoshift procedure was used to align NMR spectra in MATLAB (R2012a; The MathWorks, Inc., Natick, MA, USA) (66). The spectral region from 0.0 to 9.0 ppm excluding residual water signal (4.7 to 5.2 ppm) was subdivided and integrated to binning data with a size of 0.002 ppm for multivariate analysis.

Metabolite signals in the NMR spectra were identified by combining the Chenomx NMR suite, version 7.0 (Chenomx, Inc., Edmonton, AB, Canada), the Human Metabolome Database (67), and reported data (68). Then, a two-dimensional ^13^C-^1^H heteronuclear single-quantum coherence (HSQC) experiment was employed to confirm uncertain identifications. To quantify the relative concentration of a metabolite, its peak area was manually integrated with Topspin software and calculated on the basis of its peak area by reference to the TSP peak area.

### Data analysis and statistics.

In this study, we used principal-component analysis (PCA) to obtain an overview of microbial composition or metabolic pattern changes between different groups with MetaboAnalyst, version 3.0 (69). Data were subjected to Pareto scaling prior to PCA. To assess the interaction effect between sex and antibiotic on microbe and metabolite, a linear mixed-model (LMM) analysis of variance (ANOVA) was performed using SAS software (PROC MIXED procedure in SAS, version 9.2 [SAS Institute, Inc., Cary, NC, USA]). In the LMM model, sex (*S*), antibiotic (Abx) and their interaction (*S* × Abx) were set as fixed effects, and the individual and model intercept were as set as random effects, as given in equation 1:

(1) $$M={\text{α}}_{1}(S+{\text{β}}_{1})(\text{Abx}+{\text{γ}}_{1})(S\times \text{Abx})+\text{ε}$$

where *M* is microbial or metabolic data, the parameters *α*_1_, β_1_, and *γ*_1_ represent model coefficients, and *ε* is the random effect.

Microbial or metabolic data were calculated by a least-squares (LS) means procedure and presented as LS-means and standard error (LSM ± SE). Pairwise multiple comparisons were analyzed using Student's *t* test with a Bonferroni adjustment, and a Bonferroni-adjusted *P* value of less than 0.05 was considered statistically significant. Microbial composition data were illustrated as a heat map, and cluster analysis was performed by Ward’s method and Euclidean distance using MetaboAnalyst, version 3.0. The relationship between different microbes or between microbe and metabolite was analyzed by Pearson’s correlation, and the corresponding *P* value was calculated by the MATLAB function corr (version R2012a). The correlation network was visualized with Cytoscape software (version 3.6.0) (70). Network topological parameters were then analyzed using the NetworkAnalyzer plug-in in Cytoscape (71).

### Data availability.

All data used in this study are publicly available on our lab website (http://nmrlab.yxy.wmu.edu.cn/info/1034/1555.htm) under the appurtenant materials, “Metabolome Dataset-2019-5-8” for NMR-based metabolomics data and “Microbiome Dataset-2019-5-25” for 16S rRNA gene sequence data.
